# CT Scans in Young People in Great Britain: Temporal and Descriptive Patterns, 1993–2002

**DOI:** 10.1155/2012/594278

**Published:** 2012-06-26

**Authors:** Mark S. Pearce, Jane A. Salotti, Nicola L. Howe, Kieran McHugh, Kwang Pyo Kim, Choonsik Lee, Alan W. Craft, Amy Berrington de Gonzaléz, Louise Parker

**Affiliations:** ^1^Institute of Health & Society, Newcastle University, Sir James Spence Institute, Royal Victoria Infirmary, Newcastle upon Tyne NE1 4LP, UK; ^2^Great Ormond Street Hospital for Children NHS Trust, London WC1N 3JH, UK; ^3^Department of Nuclear Engineering, Kyung Hee University, Gyeonggi-do 446701, Republic of Korea; ^4^Radiation Epidemiology Branch, Division of Cancer Epidemiology and Genetics, National Cancer Institute, Bethesda, MD 20892, USA; ^5^Northern Institute of Cancer Research, Newcastle University, Sir James Spence Institute, Royal Victoria Infirmary, Newcastle upon Tyne NE1 4LP, UK; ^6^Departments of Medicine and Paediatrics, Population Cancer Research Program, Dalhousie University and Cancer Care Nova Scotia, Halifax, NS, Canada B3H 3B7

## Abstract

*Background*. Although using computed tomography (CT) can be greatly beneficial, the associated relatively high radiation doses have led to growing concerns in relation to potential associations with risk of future cancer. Very little has been published regarding the trends of CT use in young people. Therefore, our objective was to assess temporal and other patterns in CT usage among patients aged under 22 years in Great Britain from 1993 to 2002. *Methods*. Electronic data were obtained from the Radiology Information Systems of 81 hospital trusts within Great Britain. All included patients were aged under 22 years and examined using CT between 1993 and 2002, with accessible radiology records. *Results*. The number of CT examinations doubled over the study period. While increases in numbers of recorded examinations were seen across all age groups, the greatest increases were in the older patients, most notably those aged 15–19 years of age. Sixty percent of CT examinations were of the head, with the percentages varying with calendar year and patient age. *Conclusions*. In contrast to previous data from the North of England, the doubling of CT use was not accompanied by an increase in numbers of multiple examinations to the same individual.

## 1. Introduction

Since entering clinical service use in the 1970s as an alternative to standard X rays and ultrasound, examinations using computed tomography (CT) have rapidly become an indispensable, sometimes life-saving diagnostic tool, with ever increasing clinical applications. 

Whilst the immediate benefit to the individual patient of having a CT examination can be substantial, the relatively high radiation doses associated with CT have given rise to growing concerns from a public health perspective, in particular, due to a possible increase in future cancer risk, with estimates of increased risks of number of different cancers shown when using extrapolations from likely doses to the risk estimates produced on studies of the atomic bomb survivors [1–9]. Children can receive higher radiation doses than necessary if examined using adult CT settings [10]. Surveys have estimated that children under 15 years of age make up between 3% and 11% of patients who undergo CT examinations in Western Europe and North America [11–13]. Younger patients are more susceptible to the effects of radiation, in part due to their longer postirradiation life expectancy and because following the same radiation exposure they experience greater radiation-induced tissue damage than adults due to their increased vulnerability of rapidly dividing cells [2]. The concerns regarding CT in young people, coupled with the fact that few empirical data have been used in existing risk predictions, make this an area of epidemiological research of great importance. Further, most prediction models and previous studies of trends in CT usage have been based on adult patients, whereas the greatest concern is for children. In order to better ascertain likely risks associated with radiation exposures to young people due to CT, a large cohort study of individuals first examined with CT before 22 years of age in the United Kingdom is underway, with the primary objective of assessing the potential risks associated with radiation exposures due to CT examinations in young people. In the UK, the overall frequency of CT examinations increased by 39% between 1997/1998 and 2001/2002, whereas the frequency of conventional radiographic examinations increased by only 1% during the same period [14]. By 2008, the number of CT examinations in England alone (population 51 million) had risen to over 3 million per year [15], but age at exposure and, hence, paediatric trends data were not available. In the first descriptive paper from this study, we reported on temporal trends and patterns in CT usage in the Northern Region of England [16]. We demonstrated that the frequency of CT examinations in this age group in the North of England more than doubled over the study period and that this was, at least partly, explained by the increase in the number of examinations per patient. In this paper, we extend this analysis to all participating hospitals in Great Britain to assess the changes in CT usage over time and the way in which usage varies in relation to age, sex, and type of CT examination. 

## 2. Methods

This study covers patients in Great Britain (England, Wales, and Scotland). The participating organizations vary in the size of geographical area covered, number and type of patients treated, number and type of hospitals, the radiology departments' patient load and services provided, and public health and clinical responsibilities. 

Electronic data, from Radiology Information Systems (RIS), were obtained for patients who had had CT examinations in any of the participating National Health Service (NHS) Trusts (England and Wales) and NHS Boards (Scotland). Participating Trusts and Health Boards, from here on all called “Trusts”, were contacted to determine the availability of the data required and their willingness to contribute to the study. The retrieved data included patient identifiers, dates of birth and CT examinations, sex, postcode, and the types of CT examinations. Patient identifiers were used to identify patients having multiple procedures within a Trust, while matching on patients' names, sex, and dates of birth was used to identify patients examined at more than one participating Trust.

The data included in this study cover the ten-year period from 1993 to 2002 for patients under the age of 22 years at the first CT examination of any kind recorded on RIS. Where the installation of a RIS took place midway through a year, a pro rata adjustment was used to estimate the total number of examinations in that year. Where RIS data were missing for early years of the study period, the number of examinations in the earliest year available (with complete or pro-rata-adjusted data) was taken to apply to all previous years of CT operation in the Trust. 

Types of CT examinations were grouped into six categories (head and neck, abdomen/pelvis, chest, spine, extremities, and miscellaneous) based on those previously suggested by Mettler [17] and as used in our previous paper on the Northern Region [16]. As the number of examinations involving more than one part of the body was small, numbering 3,088 or just 1% of total examinations, they were included in the “miscellaneous” category.

These data were described in relation to temporal trends, patient ages and sex, and type of examination. Associations between categorical variables were assessed using chi-squared tests; correlations were assessed using Spearman's rank tests. All statistical analyses were done using the statistical software package Stata, version 10 (StataCorp, College Station, TX, USA).

This study, as part of the larger retrospective epidemiological cohort study, was given a favourable ethical opinion by the Newcastle and North Tyneside Local Research Ethics Committee (akin to approval) and was approved by the National Information Governance Board so as not to require individual patient-level consent. 

## 3. Results

The study contains data from 81 Trusts, including data from 12 specific children's hospitals. A total of 361,559 examination records were abstracted from RIS records for 187,614 patients under the age of 22 years between 1993 and 2002. This included 153,801 examinations (42.5%) among 81,811 female patients (43.6%), 207,284 examinations (57.3%) among 105,496 male patients (56.2%), and 474 examinations among 307 patients of unknown sex. 

Complete data were available across the entire study period for 51 Trusts. The remaining 30 Trusts were missing data for the earlier years of the study, most commonly 1993-1994 due to RIS beginning some time after the installation of a CT machine. Including the replacement of the missing number of examinations for the Trusts with missing data in the early years of the study period, the estimated numbers of patients and examinations were 203,032 and 379,262 respectively, representing 1.9 examinations per individual under 22 years of age per year over the ten-year study period. The highest number of examinations in one year for these Trusts input by the above replacement of missing data was 1462. Replacement of missing data was used only to assess temporal trends (of numbers of examinations and patients, by sex). All other descriptive statistics are based on data obtained from RISs.

**Figure 1:**
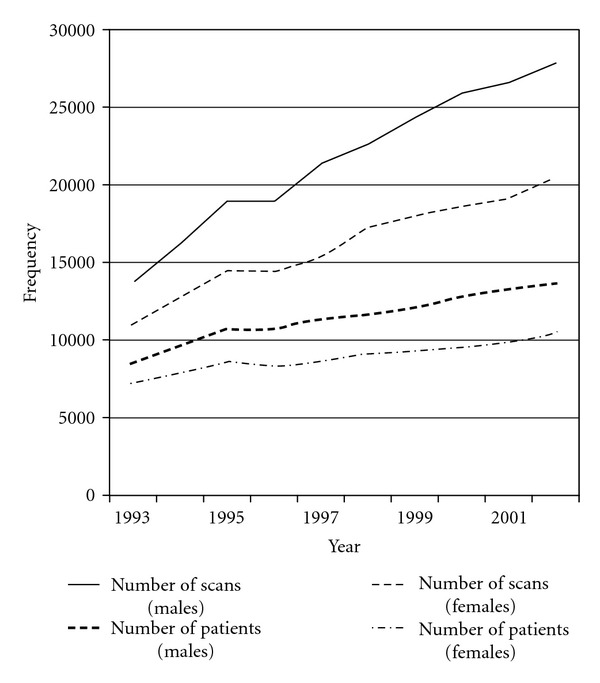
Number of examinations and number of patients per year in male and female patients under 22 years of age in Great Britain, 1993–2002.

**Figure 2:**
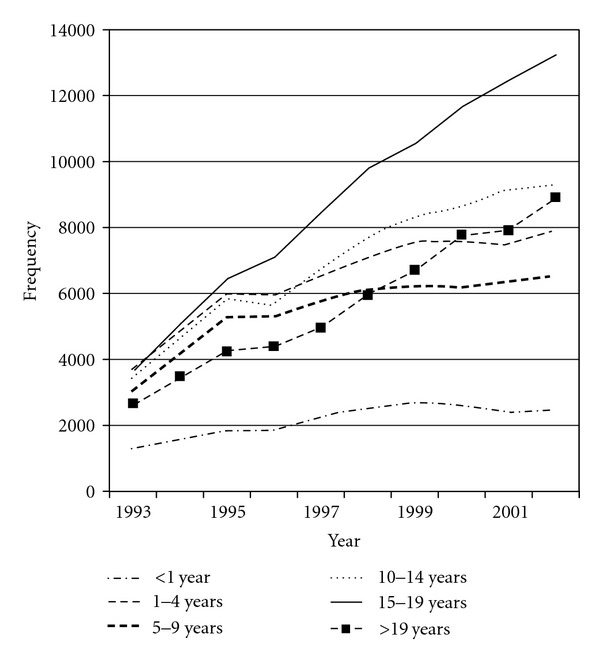
Number of examinations per year in patients under 22 years of age in Great Britain, 1993–2002, by age group (age intervals differ in size).

In the ten-year period from 1993 to 2002, the number of examinations doubled from an estimated 24,938 to 48,339 examinations per year. During the same period, the increase in the number of patients examined per year was a little less marked, rising from an estimated 15,669 patients in 1993 to 24,073 in 2002. These time trends were observed for males and females, but the rise was somewhat steeper for males (Figure 1). While increases in numbers of recorded examinations were seen across all age groups, the greatest increases over the study period were in the older patients, most notably those aged 15–19 years of age (Figure 2). There was a significant positive association between year of examination and age at the time of examination, although the correlation coefficient was small (Spearman's rho = 0.0739, *P* < 0.0001).

Sixty percent of examinations among patients under 22 years were of the head and neck (including 4970 examinations of the neck in 3116 patients), with similar proportions in males and females (Table 1). The next most common examination types were of the abdomen or chest, accounting for 12% and 9% of all examinations, respectively. The miscellaneous group (17,763 scans) included 4,226 examinations (24%) which were recorded as combinations of examinations in other groups. The majority of these (*n* = 117) were of the brain plus the cervical-spine. 

**Figure 3:**
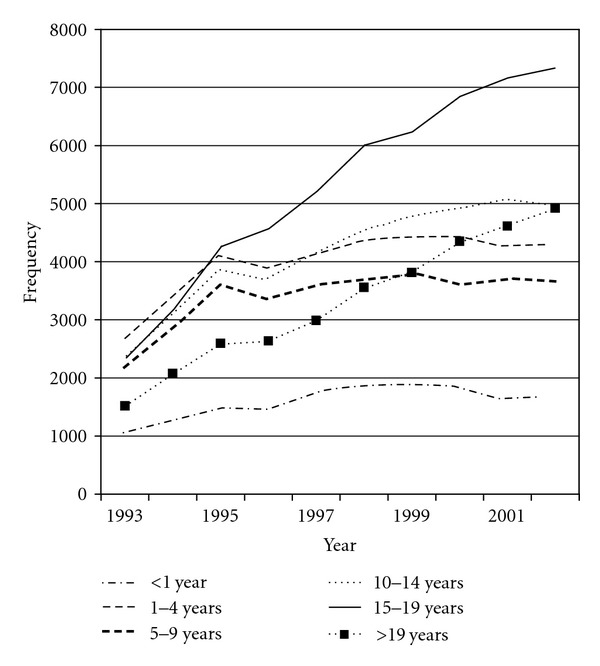
Number of head and neck CT examinations per year in patients under 22 years of age in Great Britain, 1993–2002 (age intervals differ in size).

The number of examinations per year rose similarly for both examinations of the head and other areas of the body, particularly among those aged over 15 years (Figure 3). The number of chest examinations doubled between 1995 and 1999, and similar increases were seen for examinations of the abdomen and pelvis between 1995 and 1998, with smaller increases for examinations of other sites.

There was a significant association between age at the time of the examination and the type of examination (*P* < 0.001). More than 72% of CT examinations in infants were of the head, with the percentage of head examinations falling with increasing age (Table 2). 

Four percent of patients had five or more examinations, while almost 5% of patients had three examinations (Table 3). The median number of examinations per patient per year did not rise over the study period. Between 1993 and 1997, around 70% of patients underwent one CT examination per year, with around 87% having one or two examinations and 93% having three or less. In 1999, the percentage of patients having only one examination fell to 68%, with 96% of patients having four examinations or less. 

Percentages were broadly similar for males and females across the range of numbers of examinations per patient (Table 3). In the 1,371 patients with ten or more CT examinations, 17,128 examinations (40%) were examinations of the head and neck, 8,743 (20.6%) were examinations of the abdomen or pelvis, and 5,661 (13.3%) were examinations of the chest. Similar percentages were seen for males and females separately (results not presented).

Of the 187,638 patients included in this study, 4,827 (2.6%) underwent CT examinations at more than one hospital. Seventy percent (3,384) of these patients were examined at three different hospitals, and 1,653 (34%) were examined at five different hospitals. Of these patients who were examined at more than one hospital, 64% moved between NHS Trusts as well as hospitals. Of patients examined at more than one Trust, the median number of examinations per patient during the study period was three, with 30% having two examinations and 23%, 12%, and 9% having three, four, and five examinations, respectively.

At the beginning of the study period in 1993, it is estimated that there were 52 CT machines in 37 of the participating Trusts. By 1997, this had risen to an estimate of 70 machines in 47 trusts. By the end of the study period in 2002, it is estimated that there were 89 machines in 53 Trusts. It was not possible however to obtain accurate machine information for every Trust or individual hospital that participated in this study, and data on numbers of machines used over time was only partly or fully available for 54 Trusts.

## 4. Discussion

While a number of previous publications have described trends in the use of CT, these have primarily been in adult or total populations with no breakdown by age group [14, 18, 19]. Very little has been published regarding temporal trends and other patterns of CT usage in young patients. This study of electronic data from radiology departments suggests that the use of CT in young people in Great Britain has increased over the study period of 1993–2002. In our previous analysis centered on the North of England only, the greatest increase in the annual number of examinations was between 1997 and 2000, with a concurrent increase in the median number of examinations per patient per year over the same time period [16]. However, when analysing data from across Great Britain, a more linear increase was seen in number of examinations per year and there was no increase in the average number of examinations per patients per year. The reasons for this are unclear but may include the fact that CT was introduced in different centres at different times or due to differing methods in recording multiple examinations.

As in our previous paper [16], the most common type of CT examination was of the head, particularly in infants. The number of head examinations changed over time in line with the overall increase in number of examinations per year, as did the number of examinations of other sites. The temporal trends in the use of CT in young people whose records were included in this study broadly mirror those seen in overall populations in the UK [14] and elsewhere [18]. The general increase seen in CT usage partly reflects the increasing availability and the nature of the technology. It is a rapid, relatively simple, and, most importantly, accurate diagnostic tool. The increasing speed in which CT examinations can be completed makes the modality especially applicable to very young children who would otherwise require sedation or anesthesia to keep them motionless for long enough to obtain diagnostic images. This primarily makes it more feasible to use than magnetic resonance imaging, although plain X ray is quicker still but not as sensitive as CT.

The participating Trusts ranged from national and regional centers as well as teaching hospitals to numerous regionally based acute Trusts. Although data were not totally complete over the entire study period, they were complete for the majority of included Trusts. The method chosen to estimate missing data was likely to be one of the most conservative options in terms of assessing increases in numbers of examinations. By replacing missing numbers of examinations with the number in the earliest year available for the Trust, we believe that this will have overestimated the number of examinations for the Trusts with missing data for the early years of the study period (given the increasing usage of CT over the study period) and hence reduced the magnitude of the increase in numbers of examinations and patients over time. However, as the Trusts with missing data tended to be those with relatively small numbers of CT examinations in the later years, any overestimation is likely to be small and would make little difference to the overall trends for Great Britain as a whole. Trusts in the Northern Region of England which were included in the previous paper [16] accounted for only 11% of patients in this Great Britain-wide study.

We were able to include data on examination type, age group, or sex and chose to only replace the missing data for analysis by year of examination. As these are the only data available to this study, there were no further variables that could be included to identify further patterns. Indications for examinations were not available from electronic records and, therefore, unavailable to this study. Although we are not able to include population data as the catchment areas for hospitals do match UK census data, we included nearly half of all the hospital Trusts in existence in Great Britain in 2002, with wide geographical coverage to increase the likelihood of our study being representative of the population of Great Britain. The lack of population data precludes the calculation of rates of CT usage per capita. However, in our previous paper on the North of England [16], where complete geographical coverage allowed population and rate estimates, we were able to show that the number of patients, scanned at least once, per year rose from 2.24 to 3.54 per 1000 population aged less than 22 years between 1993 and 2002.

Increases in CT examination usage for young patients have been seen in other studies, although data are sparse and in some cases limited to certain types of CT examination. Between 1996 and 1999, the number of abdominal and pelvic CT examinations among children nearly doubled in a major US hospital [2]. Similarly, our previous paper [16] reported that the number of examinations administered to those aged under 22 years of age more than doubled between 1993 and 2002. Markel et al. demonstrated an increased use of CT for pediatric blunt chest trauma in one American trauma center between 2001 and 2005 [20]. A similar increase was seen in this study, although we are able to demonstrate increases beginning further back in time. A tripling in the number of patients aged 0–18 years undergoing CT examinations between 1986 and 2008 was reported in a study of Australian CT records by Brady et al. [21]. Brady et al. demonstrated that most examinations were performed in those aged over 15 years and that the increase in examinations over time was also higher in this age group. This concurs with the findings of this study on patients in Great Britain although the age groupings between studies differ slightly. Our upper age limit of 21 years ties in with the upper age limit of first scan in our epidemiology study and allows trends and patterns to be assessed in both children and young adults simultaneously. 

It is likely that changes in clinical practice will have been behind most of the increased usage. Various national guidelines and recommendations have advocated the use of CT examinations in young patients, particularly in relation to head injuries, including three published articles shortly after the period of the largest increase in CT use in Great Britain as observed in this study [22–24]. There has been a move to CT, in the United Kingdom, from plain skull X ray for head trauma, as well as a move to abdominal and chest CT for both trauma and nontraumatic pathology in these body regions. It is likely that such guidelines will have been driven by existing changes in clinical practice, so would already reflect an existing increase in CT usage. With their publication, it would then be expected that an additional increase would occur. Given the higher likely prevalence of head injuries among the older patients (aged 15–19 years) in this study, changes in guidelines may also explain the greatest increase in numbers of CT examinations being in the older patients (as indicated by the large number of head examinations in this age group), although we have no data on reason for, or indication from, the examination to be able to investigate this further.

The majority of CT examinations in this study were of the head and neck which is similar to the examination type distribution reported in a study of young patients in Israel [7]. This is in contrast to studies of populations including all age groups which report that examinations of the abdomen and pelvis are more common, suggesting differing uses of CT in older populations [11, 25]. A study of usage patterns by age group (0–24 years) in the United States found slightly lower percentages of head examinations (50% in females and 55% in males), but higher percentages of CT being used for examinations of the abdomen and/or pelvis (28% in females and 20% in males) [8]. This is likely to reflect differences in clinical practice between the two countries. Although outside the years covered by this study, a survey of pediatric CT practices in Germany between 2005 and 2006 found that just over 50% of pediatric CT examinations were of the brain [26], a smaller percentage to that for head and neck examinations in our study (62.4%, 60.14% when not including neck examinations). The age distribution in the German study was uniform, although only three groups were used, with no delineation between infancy and other ages of early childhood. In addition to concerns regarding cancer, repeated head CT examinations in children have been raised as a concern in relation to future radiation-induced cataracts [27].

Although CT usage has increased, concerns have been raised that the use of CT is not always justified in young patients [3, 4, 28–31]. In particular the uses of multiple CT examinations to manage trauma [32–34], seizures [35, 36], chronic headaches [37], suspected appendicitis [38], and disc syndromes [30] in children have all been previously questioned. The numbers of multiple examinations per patient may have increased if patients moving between included Trusts were examined at both. However, given that only 3,095 (1.6%) of all patients moved between Trusts participating in this study, this cannot explain the increase in either total number of examinations per year or in numbers of multiple examinations per year. This rate may be an underestimate as not all hospitals in Great Britain were included in this study, due to a range of factors. This was primarily the unavailability of required RIS data, feasibility of data collection from smaller hospitals, and hospitals declining to participate in the study. 

With so little known about the use of CT in young populations around the world, it is important to report such trends and patterns, as in this report of data collected up to 2002 in our ongoing epidemiology study. Even less is known about the use of CT in this population up to the present day, in particular in relation to changes in the use of other modalities. It has been reported that the total number of CT imaging investigations in England rose from 1.1 to 2.7 million between 1996 and 2007 [39]. In contrast the number of MRI investigations rose from 0.3 to 1.3 million and ultrasound from 4.5 to 6.7 million. Therefore, our findings may not reflect the increasing use of MRI and ultrasound in more recent years nor the growing awareness of CT-related cancer risks which may have reduced CT usage in this population.

## 5. Conclusion

CT usage in this young population doubled between 1993 and 2002. The majority of examinations performed in this age group were of the head, and the highest number of head examinations was administered to those aged over 15 years. The use of CT is likely to continue to grow as technology progresses and other clinical applications emerge, such as cardiac CT, whereby CT examinations are even more efficient and cost-effective. There needs to be careful consideration of the benefits of using CT for patients, especially pediatric patients versus the risks that may be associated with the radiation exposures that these patients receive. Our ongoing epidemiological study into the long-term health effects of using CT in young people, in which patients are being matched to national-level cancer registry data, will provide the first risk estimates based on empirical data. These improved estimates are a prerequisite for future decision making.

## Figures and Tables

**Table 1:** Numbers of CT examinations by sex and type of examination in patients under 22 years of age in Great Britain, 1993–2002.

Categories	All	Males	Females
	*N* (%)	*N* (%)	*N* (%)
Head and neck	218,091 (60)	125,295 (60)	92,424 (60)
Abdomen and pelvis	42,226 (12)	24,020 (12)	18,169 (12)
Chest	31,074 (9)	17,853 (9)	13,200 (9)
Unknown	24,039 (7)	13,668 (7)	10,367 (7)
Extremities	17,853 (5)	10,559 (5)	7,285 (4)
Spine	10,513 (3)	6,493 (3)	3,999 (3)
Miscellaneous	17,763 (5)	9,396 (4)	8,357 (5)

Total	361,559 (100)	207,284 (100)	153,801 (100)

Sex not known for 474 examinations (372 “head,” 37 “abdomen,” 21 each for “chest” and “spine,” 9 “extremities,” 4 “unknown,” and 10 “miscellaneous”).

**Table 2:** Numbers of CT examinations by age and type of examination in patients under 22 years of age in Great Britain, 1993–2002.

	Age at time of examination (years)					
Categories	<1	1–4	5–9	10–14	15–19	20-21
	*N* (%)	*N* (%)	*N* (%)	*N* (%)	*N* (%)	*N* (%)
Head & neck	15,921 (73)	40,056 (61)	34,117 (61)	41,539 (59)	53,191 (59)	33,128 (57)
Abdomen & pelvis	1,156 (5)	6,260 (10)	5,080 (9)	6,979 (10)	12,491 (14)	10,230 (18)
Chest	1,739 (8)	5,430 (8)	5,052 (9)	5,823 (8)	8,019 (9)	4,995 (9)
Unknown	1,773 (9)	8,429 (13)	4,916 (9)	4,071 (6)	3,292 (4)	1,551 (3)
Extremity	471 (2)	1,679 (3)	2,352 (4)	5,646 (8)	5,212 (6)	2,485 (4)
Miscellaneous	637 (3)	2,925 (5)	3,253 (6)	4,236 (6)	3,880 (4)	2,815 (5)
Spine	120 (1)	758 (1)	1,159 (2)	2,047 (3)	3,931 (4)	2,494 (4)

Total	**21,817 (100)**	**65,537 (100)**	**55,929 (100)**	**70,341 (100)**	**90,016 (100)**	**57,698 (100)**

Age not known for 221 examinations (139 “Head”, 34 “Abdomen”, 20 “Chest”, 11 “Miscellaneous”, 8 “Extremities”, 5 “Unknown” and 4 “Spine”).

**Table 3:** Numbers of multiple examinations per patient, by sex.

No. of examinations	Total patients	Males	Females
	*N* (%)	*N* (%)	*N* (%)
1	132,962 (71)	75,658 (70)	59,061 (72)
2	34,419 (18)	19,682 (19)	14,691 (18)
3	8,837 (5)	5,308 (5)	3,511 (4)
4	4,822 (3)	2,888 (3)	1,923 (2)
5	2,113 (1)	1,260 (1)	849 (1)
6	1,362 (0.7)	811 (0.8)	549 (0.7)
7	764 (0.4)	474 (0.5)	287 (0.4)
8	614 (0.3)	380 (0.4)	232 (0.3)
9	374 (0.2)	217 (0.2)	157 (0.2)
10	294 (0.2)	176 (0.2)	118 (0.1)
11–20	928 (0.5)	554 (0.5)	372 (0.5)
>20	149 (0.1)	88 (0.1)	61 (0.1)

Total	187,638 (100)	105,496 (100)	81,811 (100)

331 patients were of unknown sex (243 had one examination, 46 had two examinations, 18 had three examinations, 11 had four examinations, and no patients of unknown sex had over 20 examinations).
